# Stuck with a CRPS patient - what else to offer a CRPS patient?

**DOI:** 10.1186/1753-6561-9-S3-A84

**Published:** 2015-05-19

**Authors:** Mary Cardosa

**Affiliations:** 1 Department of Anaesthesia, Hospital Selayang, Selangor, 68100, Malaysia

## 

Complex regional pain syndrome or CRPS includes painful conditions characterised by persistent pain, confined to a particular region of the body that is seemingly disproportionate to the initiating injury. The pain is usually associated with any of the following: abnormal sensory, motor, sudomotor, vasomotor or trophic changes, and shows variable progression over time. Many CRPS patients have severe pain and disability as well as impact on sleep and mood, and are often challenging to manage.

Management must begin with a thorough assessment of symptoms and signs as well as the psychosocial impact of the pain. The diagnosis of CRPS is usually a clinical one with no specific confirmatory tests required, and treatment should take a multidisciplinary, multimodal approach, with the overall aim of improving not just pain but more importantly, function and mood. Modalities of treatment include medications, interventions (sympathetic nerve blocks), physical therapy and psychological therapy.

Appropriate medications include antineuropathic agents including tricyclic antidepressants, serotonin-noradrenaline reuptake inhibitors (SNRIs) and anticonvulsants like the alpha2delta ligands. Exercise and rehabilitation with aim of return of function is a crucial part of the management; often, pain prevents the patient from doing effective exercise, and use of opioid medications (weak opioids like tramadol or even strong opioids like oxycodone) may be necessary to enable patients to start their physical therapy. Other modalities of treatment include Mirror therapy and sympathetic blocks (for patients with sympathetically maintained pain). All patients must also be taught to do relaxation and to use it to manage their pain. Patients who are very disabled by their pain, especially those whose pain does not decrease with appropriate medications, may require cognitive behavioural interventions including intensive pain management programs which teach patients how to function normally *despite* persistent pain.

Our experience in Selayang hospital with patients with difficult-to-manage CRPS will be discussed in the presentation.

